# Early Use of Cardiac Resynchronization Therapy to Accelerate Symptomatic Relief and Complete Left Ventricular Function Recovery in Peripartum Cardiomyopathy

**DOI:** 10.3390/medicina55060246

**Published:** 2019-06-05

**Authors:** Elizabeth Richard, Pierre Yves Turgeon, Michelle Dubois, Mario Sénéchal

**Affiliations:** 1 Research Center, Institut Universitaire de Cardiologie et de Pneumologie de Quebec, Laval University, Quebec, QC G1V 0A6, Canada; elizabeth.richard.1@ulaval.ca (E.R.); michelle.dubois@criucpq.ulaval.ca (M.D.); 2 Department of Cardiology, Institut Universitaire de Cardiologie et de Pneumologie de Quebec, Laval University, Quebec, QC G1V 0A6, Canada; pierre-yves.turgeon.1@ulaval.ca

**Keywords:** peripartum cardiomyopathy, CRT, heart failure, remodeling

## Abstract

Peripartum cardiomyopathy (PPCM) is a rare cause of heart failure that develops during the last month of pregnancy or within first months of delivery. We report the case of a 40-year-old woman diagnosed with severely symptomatic PPCM characterized by left ventricular ejection fraction (LVEF) of 10% and significant dyssynchrony secondary to a left bundle branch block (LBBB). Early cardiac resynchronization therapy (CRT) was used to achieve remarkable functional and LVEF recovery. This case suggests that early CRT must be considered for patients suffering from severely symptomatic PPCM despite optimal medical therapy for whom advanced heart failure therapies are proposed.

## 1. Introduction

Peripartum cardiomyopathy (PPCM) is a rare and idiopathic cause of heart failure that develops during the last month of pregnancy or within first months of delivery. As any heart failure with reduced left ventricular ejection fraction (LVEF), a left ventricular assist device (LVAD) or a heart transplant must be considered in severely symptomatic patients despite optimal medical therapy. Less than 5% of patients suffering from PPCM will have an enlarged QRS width but this characteristic must not be overlooked as some could benefit from cardiac resynchronization therapy (CRT) [1].

## 2. Case Report

**Figure 1:**
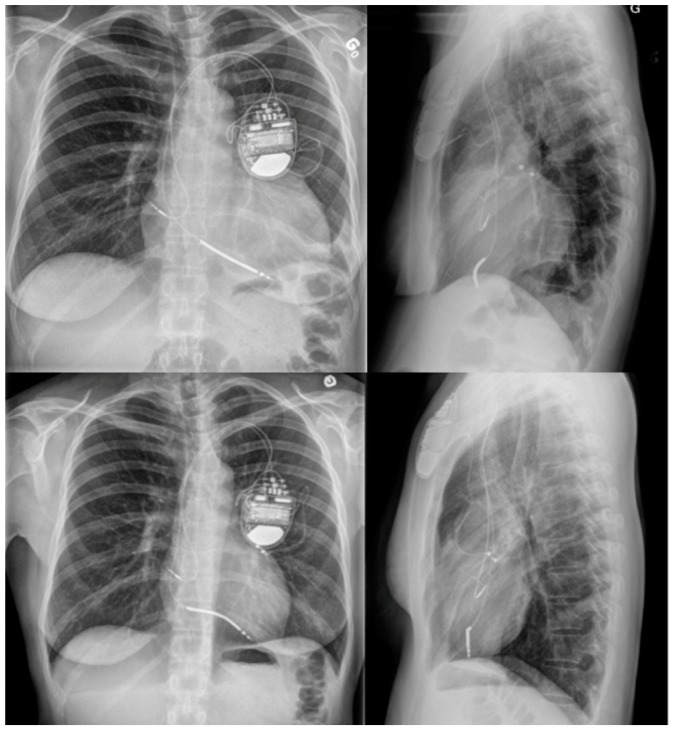
Chest X-rays imaging CRT leads position and increased cardio-thoracic index immediately after implantation (upper line). Remarkable normalization of cardio-thoracic index after 13-month follow-up (lower line).

A 40-year-old woman (gravida 4, para 4) was evaluated at our specialized center for rapidly progressive symptoms of heart failure in the first week postpartum. The patient’s medical background and first three pregnancies were unremarkable. The last pregnancy was uneventful except for a caesarian section for nuchal cord during a term spontaneous labor. She was prescribed antibiotics on day five postpartum for new onset dyspnea and dry coughing. She rapidly deteriorated with dyspnea at rest, orthopnea, and paroxysmal nocturnal dyspnea. Echocardiography showed a left ventricular ejection fraction (LVEF) of 10%, severe left ventricular dilatation with left ventricular end-systolic diameter (LVESD) of 58 mm and moderate functional mitral regurgitation. The electrocardiogram showed sinus rhythm and new onset left bundle branch block (LBBB) with a QRS width of 158 ms. Dobutamine perfusion was initiated for low cardiac output signs along with intravenous Furosemide. The coronary angiogram was normal and the Swan–Ganz catheterization showed a cardiac index of 1.71 L/min on inotropic support with normal pulmonary artery and capillary wedge pressures. The cardiac MRI showed no sign of inflammation or fibrosis. At this point, the differential diagnosis included peripartum cardiomyopathy and idiopathic dilated cardiomyopathy. However, the latter seemed less likely because of the absence of previous symptoms and the acute onset of symptoms in the early postpartum period, which is characteristic of peripartum cardiomyopathy. Valsartan 40 mg PO twice a day and Eplerenone 25 mg PO daily was initiated. Slow weaning of Dobutamine was completed after five days and small dose Metoprolol was eventually added. Despite uptitration of therapy, the patient did not improve. Bromocriptine was discussed with the patient considering some evidence to support its use but was not initiated because of the initial preference of the patient to breastfeed. However, the inherent difficulties of the long hospitalization led the patient to abandon breastfeeding [2]. At this point, complete pre-transplant assessment was performed. Considering the typical LBBB and enlarged QRS > 150 ms, we decided to implant CRT. An endovenous dual chamber pacemaker and defibrillator was implanted but unfortunately, due to unfavorable anatomy and ostial stenosis of the two posterolateral branches, the left ventricular pacing lead implantation failed. Considering the possible benefits and absence of significant clinical improvement, a minimally invasive thoracotomy was performed to implant a left ventricular pacing lead (Figure 1).

**Figure 2:**
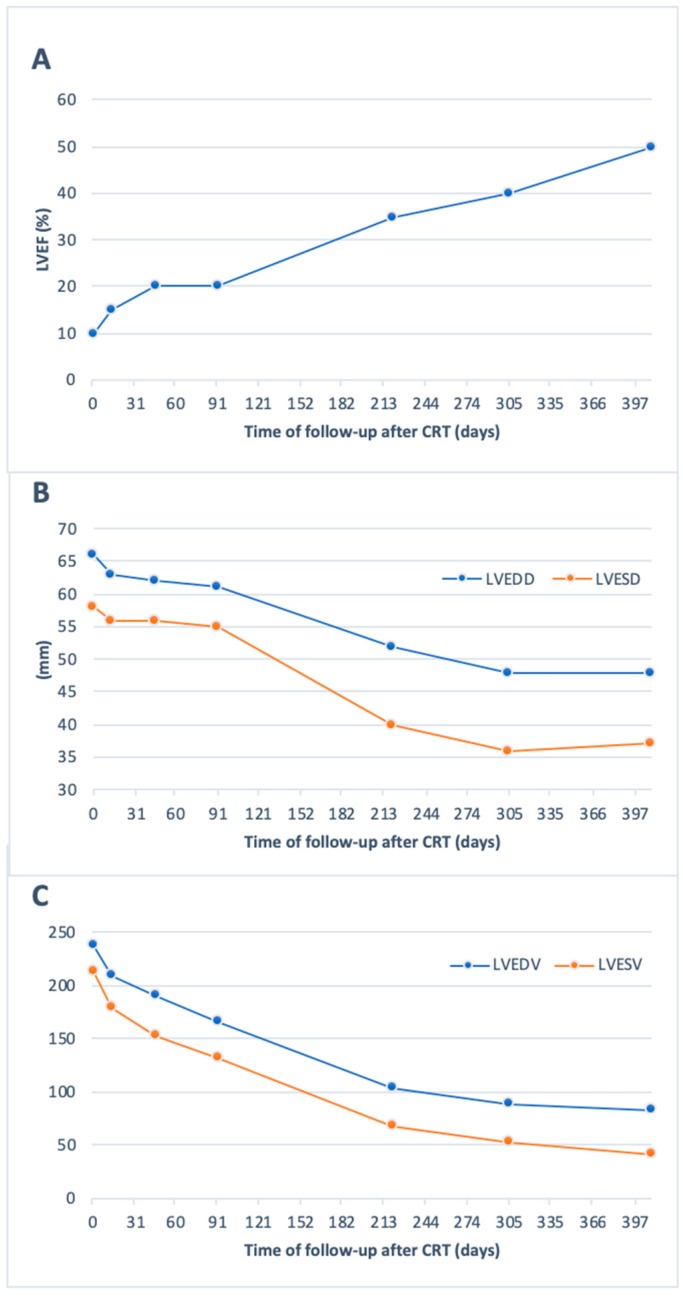
Echocardiographic parameters evolution over 13 months of follow-up. LVEF, left ventricular ejection fraction; LVEDD, left ventricular end-diastolic diameter; LVESD, left ventricular end-systolic diameter; LVEDV, left ventricular end-diastolic volume; LVESV, left ventricular end-systolic volume.

After the intervention, the patient improved to a NYHA functional class II. Control echocardiography showed resolution of mitral regurgitation and a mild increase in LVEF of 15%. The patient was followed at our specialized heart failure outpatient clinic and the titration of the optimal medical therapy was done including Sacubitril-Valsartan up to 49–51 mg PO twice a day, Bisoprolol 10 mg PO daily, and Aldactone 25 mg PO daily. The echocardiography follow-up showed increase in LVEF to 35% and 50% after seven and thirteen months respectively (Figure 2).

## 3. Discussion

Treatment of PPCM is mainly based on guidelines directed optimal medical therapy but studies with specific treatments, like Bromocriptine and Pentoxifylline, suggest some benefits [2]. Although the etiology of PPCM is still unknown, in recent years a number of contributory mechanisms have been recognized to initiate and propagate the disease. Serum levels of a fragment of prolactin increase during the puerperal period. In women who develop PPCM, the 16 kDa prolactin fragment is overexpressed and is thought to initiate and perpetrate excessive oxidative stress which culminates in myocardial dysfunction and symptomatic heart failure [2]. In a retrospective study of 100 patients, only 23% had complete recovery, defined by an LVEF ≥ 50%, over a mean of 26 months [1]. Of those, a single patient had a baseline LVEF <20%. Only 4% had QRS width >120 ms and 2% eventually benefited from CRT.

In the IPAC study, no patient with both baseline LVEF < 30% and an LVEDD ≥ 60 mm had complete recovery at 12 months [3]. As our patient had both characteristics on baseline, the IPAC study results were the rationale for our decision to implant a defibrillator instead of a pacemaker only. Mouquet et al. have described impressive responses to CRT for late the recovery of LVEF in PPCM but, according to published cases and series, the present case is the first describing an early use of CRT [4]. With a LBBB and QRS width of over 150 ms, the patient had the two unequivocal criteria for CRT as stated by the major international guidelines [5]. Moreover, female gender and non-ischemic cardiomyopathy are major predictive factors to identify CRT responders. Among responders to CRT, there is a proportion of patients who are referred to as super-responders since their left ventricular function improves to normal or near-normal values. Previous clinical studies in patients with symptomatic heart failure have reported an incidence of 10%–29% of CRT hyper-responders (commonly defined as improvement of LVEF ≥ 50% with functional recovery (NYHA class II or II)) [5]. The time to recovery in super-responders to CRT has been variable among patients and was usually between 3 and 6 months. Few studies have assessed left ventricular reverse remodeling over a very long period (≥12 months) [6]. Since the patient we described recovered a normal LVEF and improved to a NYHA functional class II after CRT implantation over a period of 12 months, we can identify her as a hyper-responder.

## 4. Conclusions

In conclusion, in presence of LBBB with severe systolic dysfunction, early CRT should be considered to accelerate functional recovery in patients with PPCM that are evaluated for advanced support therapies considering that the other options are far more invasive and morbid.
